# The Effect of *Hydrocotyle sibthorpioides* Lam. Extracts on *In Vitro* Dengue Replication

**DOI:** 10.1155/2015/596109

**Published:** 2015-02-12

**Authors:** Fitrien Husin, Yean Yean Chan, Siew Hua Gan, Siti Amrah Sulaiman, Rafidah Hanim Shueb

**Affiliations:** ^1^Department of Medical Microbiology and Parasitology, Universiti Sains Malaysia, Kubang Kerian, 16150 Kelantan, Malaysia; ^2^Human Genome Centre, Universiti Sains Malaysia, Kubang Kerian, 16150 Kelantan, Malaysia; ^3^Department of Pharmacology, Universiti Sains Malaysia, Kubang Kerian, 16150 Kelantan, Malaysia

## Abstract

*Objective*. To investigate the potential effect of *Hydrocotyle sibthorpioides* Lam. (*H. sibthorpioides*) extracts against *in vitro* dengue viral replication. *Methods*. The cytotoxicity of *H. sibthorpioides* was evaluated using a cell viability assay. Cells were pre- and posttreated with water and methanol extracts of *H. sibthorpioides*, and the viral inhibitory effect was investigated by observing the morphological changes, which were further confirmed by plaque assay. *Results*. The methanolic extract cytotoxicity was higher in Vero and C6/36 cells than the cytotoxicity of the water extract. Preincubation of the cells with *H. sibthorpioides* extract showed nonexistent to mild prophylactic effects. The posttreatment of Vero cells with *H. sibthorpioides* methanolic extract presented higher antidengue activities when compared with the water extract. Surprisingly, posttreatment of C6/36 cells resulted in an enhancement of viral replication. *Conclusion*. *H. sibthorpioides* had variable effects on dengue viral replication, depending on the treatment, cell lines, and solvent types. This study provides important novel insights on the phytomedicinal properties of *H. sibthorpioides* extracts on dengue virus.

## 1. Introduction

Dengue virus (DENV) is a mosquito-borne member of the Flaviviridae family and is transmitted through the bites of* Aedes aegypti* and* Aedes albopictus* female mosquitoes. Four different serotypes of DENV exist, namely, dengue serotypes 1, 2, 3, and 4 [1]. Infection with one of the serotypes may be asymptomatic but commonly results in a flu-like illness termed dengue fever, ranging to more severe forms, such as dengue hemorrhagic fever or dengue shock syndrome. The incidence of dengue has grown dramatically in the world in recent decades. It has been reported that over 2.5 billion people are now at risk for dengue infection [2]. In Malaysia, a 277% increase in cases was reported in early 2014 compared with the same period in 2013 [3]. The present treatments for patients with dengue fever tend to be more supportive than curative. Treatments include bed rest, fluid replacement, and antipyretic agents. At best, prevention lies in proper mosquito control.

Asia, with its rich flora and fauna, is one of the most promising regions for the discovery of novel biologically active substances. A large and ever-expanding global population has expressed its preference towards natural products in treating and preventing medical problems [4].* H. sibthorpioides* is a widespread uncultivated perennial herb that holds an important place in Chinese herbal medicine. Chinese people of the Hakka ethnic group use it to treat several diseases, including adenolymphitis, herpes zoster and cholecystitis. It also has some folkloric uses in the treatment of several other minor illnesses including fever and edema, in detoxication and for soothing throat pain [5]. In addition, it has been shown to act as an antidiuretic and is effective, when applied externally, for skin tumors and in enhancing phagocytic activity and immune function [6]. Another important use of* H. sibthorpioides* in traditional Chinese medicine is to treat hepatitis [7]. Recently, the* in vitro* and* in vivo* antiviral properties of* H. sibthorpioides* against hepatitis B virus replication have been demonstrated [8]. However, the antiviral effect of this plant against other viruses has not been investigated despite its folkloric use. In this study, we investigated the potential effect of* H. sibthorpioides* extracts against dengue viral replication* in vitro*.

## 2. Materials and Methods

### 2.1. Plant Materials and Extraction

Fresh whole plants of* H. sibthorpioides*, including the aerial parts, were collected and identified to be of the correct species (reference number SBID: 001/09) by botanists from the Forest Research Institute Malaysia (FRIM). The plants were washed under running tap water and finally rinsed with distilled water. The plants were then air dried for 48 h, homogenized into a fine powder and stored in air-tight plastic containers. The powder was then extracted in a soxhlet extractor for 24 h, using either methanol or water. Both types of extract were evaporated to dryness under pressure and in a controlled temperature using a rotavap (Buchi, Flawil, Switzerland). Finally, the extracts were freeze-dried and stored until use.

### 2.2. Cell Lines and Growth Conditions

Three types of cell lines were used in this study, C6/36 cells, PScloneD, and Vero cells. C6/36 cells were maintained in L-15 medium (Sigma) supplemented with 5% fetal bovine serum (FBS) at 28°C. PScloneD swine fibroblast (PS) cells were grown in L-15 medium at 37°C. Vero cells, derived from the kidney of an African green monkey, were maintained in Dulbecco's Modified Eagle Medium (DMEM) at 37°C with 5% carbon dioxide.

### 2.3. Virus Stock

Dengue virus type-2 (DENV-2) used in this study is a prototype of the New Guinea C strain, a kind gift from the Institute of Health and Community Medicine (IHCM), Universiti Malaysia Sarawak. The virus stock was prepared in T75 cm^2^ tissue culture flasks by inoculating 70–80% confluent C6/36 cells with 200 *μ*L virus stock diluted in 2 mL of medium supplemented with 1% FBS. After 1.5 h of viral adsorption, a 1% FBS complete growth medium was added and the virus was allowed to propagate at 28°C until cytopathic effects (CPE) were observed. The cells and the culture supernatant were then harvested by gentle pipetting followed by centrifugation at 1500 rpm for 10 min. The viral supernatant was collected in 1 mL aliquots and was stored at −80°C as a viral stock until further use. The virus titer was determined by plaque assay using PS cells.

### 2.4. Cytotoxicity Test

A cytotoxicity test was performed to determine the maximum nontoxic dose of the plant extracts. The cells were seeded at 2 × 10^4^ cells per well in 96-well plates. Serial two-fold dilutions of the extracts were added to the wells in triplicate with increasing concentration (2–10,000 *μ*g/mL). Cell viability was measured using the CellTiter 96 AQ_ueous_ one solution cell proliferation assay (Promega) following four days of incubation. The absorbance was measured at 490 nm using a 96-well plate reader. The percentage of living cells was calculated by comparison with healthy untreated cells.

### 2.5. Antiviral Activity

Since one dose of* H. sibthorpioides* extracts concentration may not be adequate for the DENV-2 populations, three different doses of extract concentrations were chosen to be tested from the MNTD range. Meanwhile two virus concentrations (200 and 2000 pfu DENV-2) were tested to investigate the different virus concentration on a constant extract dose.

*(a) Morphological Changes.* The antiviral properties of* H. sibthorpioides* were investigated by examining the cell morphological changes and CPE. In this study, the morphological changes in the cells were observed and noted as the first possible indication for inhibition of DENV-2 replication in the assayed treatment. The degree of CPE manifestation was assessed using a grading system as previously described by Tang et al. [9] with some modifications: “++++” equivalent to 100% virus growth-like CPE, “+++” for 75% virus growth-like CPE, “++” for 50% virus growth-like CPE, “+” for <50% virus growth-like CPE, and “−” for no CPE. No deterioration or CPE was observed in uninfected Vero and C6/36 cells.

*(b) Pretreatment of Plant Extracts on DENV-2 Infected Cells*. Different concentrations of each extract were added in triplicate to Vero and C6/36 monolayer cells in 96-well plates for 5 h. Subsequently, the extracts were removed by washing twice with PBS and the cells were challenged with either 200 or 2000 pfu of DENV-2. After viral infection for 1.5 h, the cells were washed twice with PBS to remove any residual unbound viruses and were overlaid with culture medium containing 1% FBS complete growth medium. The cells were observed daily for any morphological changes, and at day 4 postinfection, culture supernatants were collected for virus titration.

*(c) Posttreatment of DENV-2 Infected Cells with Plant Extracts*. In the posttreatment assay, Vero and C6/36 cells grown in 96-well plates were infected with either 200 or 2000 pfu of DENV-2. After 1.5 h of viral infection, the cells were washed twice with PBS to remove any residual unbound viruses. This was followed by the addition of serial dilutions of plant extracts in triplicate. Four days after infection, the supernatants were collected for titration.

### 2.6. Determination of Viral Titers

Viral titers were determined by plaque assay. PscloneD cells were first seeded in 24-well plates at a density of 1 × 10^5^ cells/well. One-hundred microliters of DENV-2 stock were added to 900 *μ*L of diluent, and the mixture was serially diluted 10-fold. One-hundred microliters of each dilution were added to each well in duplicate, and the cells were allowed to settle for 1–3 hours. Subsequently, 0.5 mL of overlay medium containing 3% FBS and 1% carboxymethylcellulose were added, and the plates were incubated for 6 days. At the end of the incubation, the overlay was removed and the plates were gently washed with PBS (twice) before addition of methylene blue in 1% formalin for 1-2 h. The plates were then washed again, and the plaques were counted and expressed as plaque forming units per milliliter (pfu/mL).

### 2.7. Statistical Analysis

Values were expressed as means ± standard errors of the mean. Significance difference, determined as *P* < 0.05, was calculated using Student's *t*-test (Microsoft Excel).

## 3. Results

### 3.1. Cytotoxicity of* H. sibthorpioides* Extracts

**Figure 1:**
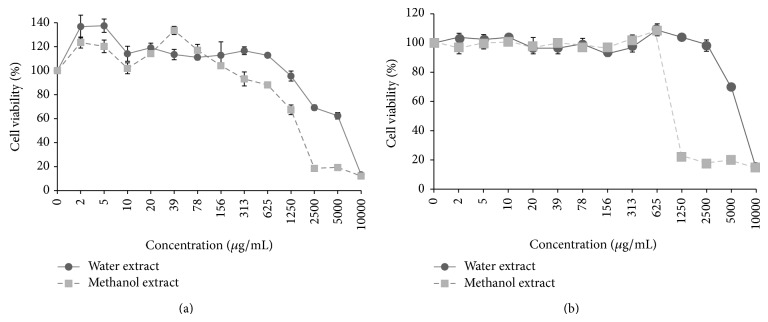
Cytotoxicity assay of* H. sibthorpioides* extracts against (a) C6/36 cells and (b) Vero cells. The assay was performed after 96 hours of treatment with various concentrations of the plant extracts. The results are presented as percentage of cell viability from triplicate assays. Bars indicate standard error.

To determine the nontoxic dose, C6/36 and Vero cells were exposed to twofold serially diluted water and methanol extracts at concentrations ranging from 2 to 10,000 *μ*g/mL (Figure 1). No signs of toxicity were observed in C6/36 cells when the water and methanol extracts were added at concentrations ranging from 2 to 625 *μ*g/mL and from 2 to 156 *μ*g/mL, respectively (Figure 1(a)). The cells showed a healthy monolayer similar to the healthy control with no loss of monolayer, and no obvious rounding, granulation, or shrinking of cells was observed. The Vero cells showed a much higher tolerance towards the dose of plant extracts when compared with C6/36 cells (Figure 1(b)). The nontoxic dose was in the range of 2 to 1250 and 2 to 625 *μ*g/mL for water and methanol extracts, respectively.

### 3.2. Antiviral Activity of Plant Extracts

The extracts were further evaluated for their prophylactic effect (pretreatment) and the ability to inhibit replication following infection of the cells with the virus (posttreatment). However, since the range of the nontoxic concentration of the extracts differed a lot (Figure 1(b)), depending on the type of cells (Vero cells had wider tolerance range) and solvent (cells were more tolerable to water extracts) used, a fixed and similar concentration of the plant extracts to be tested in both Vero and C636 cells could not be determined. Hence, within the nontoxic dose range of each cell type and solvent used, three different concentrations of the extracts were selected for further antidengue studies to examine the effect of* H. sibthorpioides* extract concentration on dengue replication: high, moderate, and low extract concentrations (Table 1). Although the extract concentrations used were different in Vero versus C636 cells, we were in part interested to study the antidengue activities when cells were exposed to the highest tolerable dose of the plant.

In addition, to study the effect of virus concentration of the possible* H. sibthorpioides* antidengue activities, cells were challenged with either 200 or 2000 pfu/mL DENV-2 following pretreatment and posttreatment of Vero and C636 cells with* H. sibthorpioides* extracts. The* in vitro* investigation of* H. sibthorpioides* on DENV-2 replication was scored by (a) observing the morphological changes in cell lines in concordance with the control and (b) measuring the difference in viral titers using plaque assay to investigate the infectivity of DENV-2.

**Figure 2:**
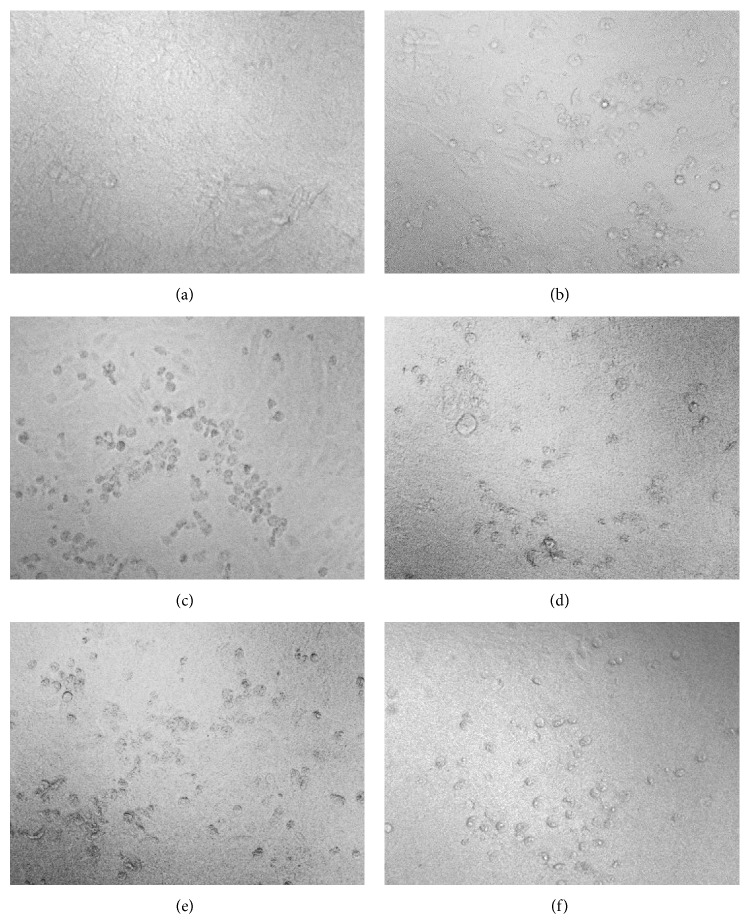
Morphological changes of Vero cells pre- and posttreated with* H. Sibthorpioides* extracts: (a) healthy noninfected cells, (b) cells infected with 200 pfu DENV-2, (c) cells infected with 2000 pfu DENV-2, (d) cells infected with 200 pfu DENV-2 and posttreated with 625 *μ*g/mL of methanolic extract, (e) cells infected with 2000 pfu DENV-2 and posttreated with 625 *μ*g/mL of methanol extract, and (f) cells pretreated with 625 *μ*g/mL water extract and infected with 200 pfu DENV-2.

**Figure 3:**
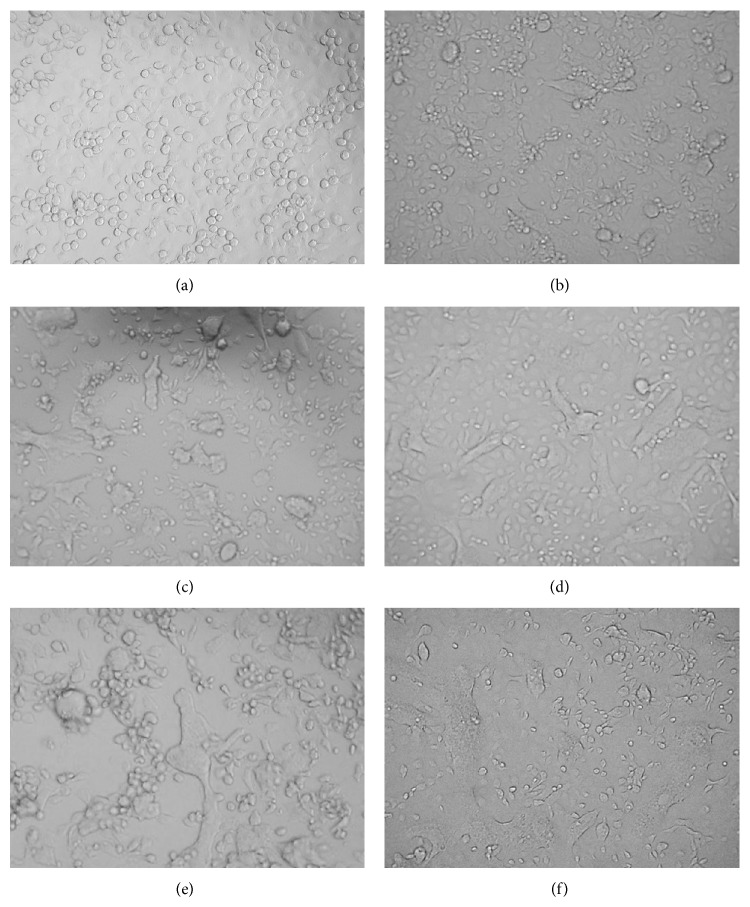
Morphological changes of C6/36 cells pre- and posttreated with* H. sibthorpioides* extracts: (a) healthy noninfected cells, (b) cells infected with 200 pfu DENV-2, (c) cells infected with 2000 pfu DENV-2, (d) cells infected with 200 pfu DENV-2 and posttreated with 625 *μ*g/mL of water extract, (e) cells infected with 2000 pfu DENV-2 and posttreated with 625 *μ*g/mL water extract, and (f) cells pretreated with 156 *μ*g/mL methanolic extract and infected with 2000 pfu DENV-2.

*(a) Prophylactic Effect of Plant Extracts on Vero and C6/36 Cells.* In DENV-2-infected Vero cells, CPE was less pronounced. In fact, lysis was only observed in certain areas of the cells, and lifting of dead cells was also noted (Figure 2). In contrast, CPE in infected C6/36 cells could be clearly identified by cell enlargement, cell fusion, syncytia, and vacuolization (Figure 3). Thus, the ability of the plant extracts to maintain the morphological state of healthy cells was a qualitative indicator of the antiviral activity.

To test for prophylactic effects of* H. sibthorpioides*, the cells were pretreated with water and methanol extracts followed by daily observations for four consecutive days. Pretreatment of Vero cells with low, moderate, and high concentrations of water (20, 156 and 1250 *μ*g/mL) and methanol (39, 156 and 625 *μ*g/mL) extracts, prior to DENV-2 infection, resulted in a CPE almost identical to positive virus growth-like CPE (approximately 75–100% CPE). The cells tended to show disintegration when compared with uninfected negative control. This finding suggests that pretreatment with the* H. sibthorpioides* extracts possibly conferred either no or low protection against DENV-2 infection.

**Figure 4:**
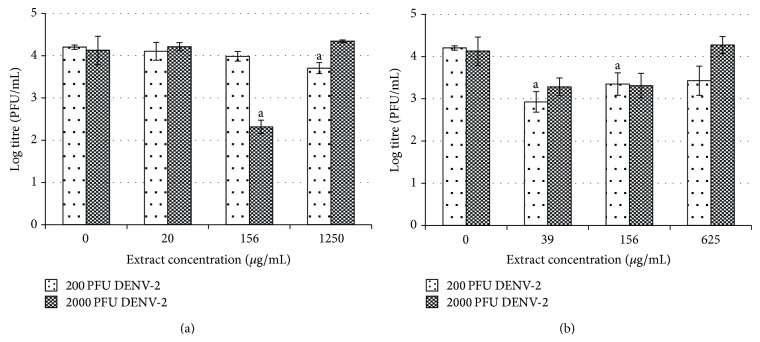
Prophylactic effect of* H. sibthorpioides *(a) water extract and (b) methanolic extract in Vero-infected cells.

The morphological changes in the treated cells were further confirmed by plaque assay. Inconsistent antiviral effects were observed when the Vero cells were pretreated with 20, 156, and 1250 *μ*g/mL of water extract (Figure 4). Following infection with 200 pfu of DENV-2, exposure to 1250 *μ*g/mL of water extract resulted in a 12% reduction of the viral titer from log 4.20 pfu/mL to log 3.71 pfu/mL (*P* < 0.05). Following infection with 2000 pfu of DENV-2, a reduction was observed only when 156 *μ*g/mL of water extract was applied (*P* < 0.05). The viral titer was reduced from log 4.13 pfu/mL to log 2.32 pfu/mL (44% reduction) (Figure 4(a)).

Interestingly, pretreatment of Vero cells with various concentrations of* H. sibthorpioides* methanolic extract generally resulted in reduced viral replication following DENV-2 infection (Figure 4(b)). In fact, following infection with 200 pfu of DENV-2, a significant viral reduction could be observed when 39 and 156 *μ*g/mL of methanolic extract were used. The viral titer was reduced from log 4.20 pfu/mL to log 2.93 and 3.35 pfu/mL. Similarly, following infection with DENV-2 at 2000 pfu, the pretreatment with the methanolic extract also had an antiviral effect, although the reduction was not statistically significant.

**Figure 5:**
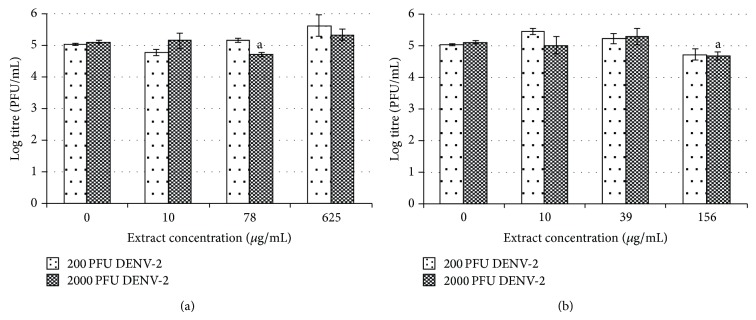
Prophylactic effect of* H. sibthorpioides *(a) water extract and (b) methanolic extract in C6/36-infected cells.

In general, the pretreatment of C6/36 cells with water and methanolic extracts of* H. sibthorpioides* did not confer any protection to the cells from DENV-2 infection (Figure 5). In C6/36 cells, positive virus growth-like CPE was observed following pretreatment of C6/36 cells with various concentrations of water and methanolic extract of* H. Sibthorpioides*, where cells were enlarged and were lysed in comparison with uninfected C6/36 cells (Figure 2). Viral replication was only significantly reduced when 78 *μ*g/mL of water extract was added to C6/36 cell prior to infection with 2000 pfu DENV-2 with less than 1 log unit reduction. The viral titer was reduced from log 5.12 pfu/mL to log 4.73 pfu/mL (8%). Nevertheless, the other investigated concentrations of water extract used in this study did not result in any significant changes.

Similarly, upon infection with DENV-2, only the pretreatment with 156 *μ*g/mL of methanolic extract showed some prophylactic effects with significant reduction of the viral titer after infection with 2000 pfu of DENV-2. The viral titer was reduced from log 5.05 pfu/mL to log 4.73 pfu/mL (6%) when 200 pfu DENV-2 was used, whereas it was reduced from log 5.12 pfu/mL to log 4.68 pfu/mL (9%) when 2000 pfu DENV-2 was used. The other concentrations of extracts used in this assay did not exhibit any prophylactic effect towards C6/36 cells.

In conclusion, water and methanolic extracts showed nonexistent to low prophylactic effects towards DENV-2 infection in C6/36 cells. Better prophylactic effects were observed in Vero cells when compared to the C6/36 cells, which is inconsistent with and independence of the virus and plant extract concentrations.

*(b) Posttreatment Effect of H. sibthorpioides Extracts on Dengue Viral Replication in Vero and C6/36 Cells.* Comparatively, a posttreatment assay was conducted to determine the antiviral activities of* H. sibthorpioides* towards DENV-2 replication. Different patterns were observed when Vero and C6/36 cells were posttreated with water and methanolic extract of* H. sibthorpioides*. In the posttreatment of Vero cells with 20, 156, and 1250 *μ*g/mL of water extract, 75% of virus growth-like CPE was observed when compared with nontreated virus control. However, in the posttreatment assay with low, moderate and high concentrations of methanol extract (39, 156, and 625 *μ*g/mL), the cells showed only 50% virus growth-like CPE. Interestingly, the methanolic extract of* H. sibthorpioides* could confer protection against DENV-2 infection, as observed by the low amount of cell death and lysis.

**Figure 6:**
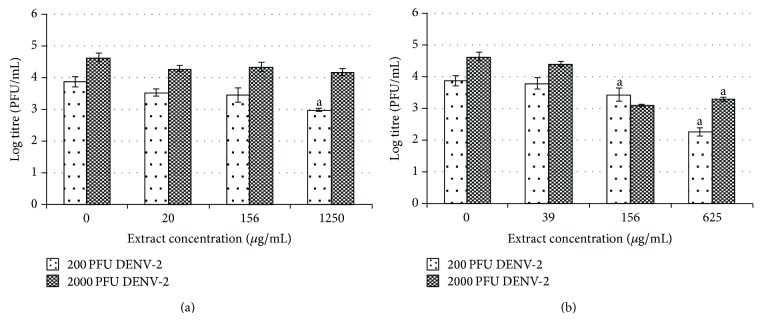
Posttreatment effect of* H. sibthorpioides *(a) water extract and (b) methanolic extract in Vero-infected cells.

The results from the plaque assay further supported the above finding (Figure 6). A dose-dependent inhibitory effect on viral replication was observed when Vero cells were posttreated with water extract after infection with 200 pfu of DENV-2. When Vero cells were posttreated with water extract at low, moderate, and high concentrations of (20, 156, and 1250 *μ*g/mL), the viral titers were reduced from log 3.88 pfu/mL to log 3.53, 3.45, and 2.98 pfu/mL, respectively. The highest (23%) inhibitory effect was observed when the highest concentration (1250 *μ*g/mL) was used. In contrast, following similar treatment in cells infected with 2000 pfu of DENV-2, comparable antiviral activities were observed, regardless of the concentration of extract used (Figure 6(a)).

A dose-dependent inhibitory effect was also demonstrated in Vero cells upon treatment with methanolic extract but with enhanced antiviral activities (Figure 6(b)). Postexposure of the cells with 39, 156, and 625 *μ*g/mL of methanolic extract reduced the DENV-2 titer from log 3.88 pfu/mL to log 3.79, 3.44, and 2.26 pfu/mL, respectively, following infection with 200 pfu DENV-2. Similarly, viral titers decreased from log 4.64 pfu/mL to log 4.41, 3.10 pfu/mL and log 3.29 pfu/mL following virus challenge with 2000 pfu of DENV-2. The greatest inhibitory effect was observed upon treatment with 625 *μ*g/mL of methanolic extract, with 42% and 29% reduction of viral titer after infection with 200 and 2000 pfu of DENV-2, respectively.

**Figure 7:**
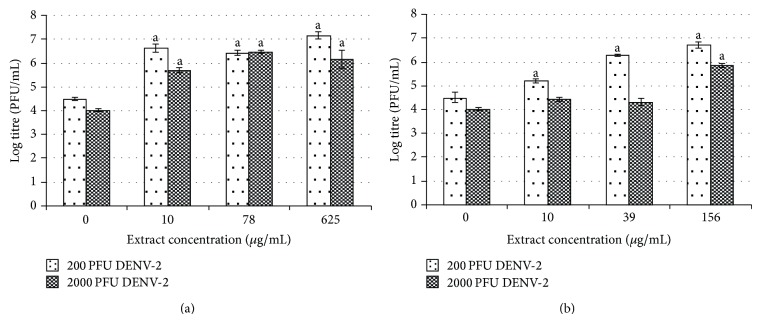
Posttreatment effect of* H. sibthorpioides *(a) water extract and (b) methanolic extract in C6/36-infected cells.

Surprisingly, microscopic observation suggested that the posttreatment with water and methanol extracts of* H. sibthorpioides* could not protect C6/36 cells from DENV-2 infection. Posttreated cells had 100% virus growth-like CPE following DENV-2 infection (Figure 2). In fact, the posttreatment with water and methanol* H. sibthorpioides* extracts in C6/36 cells resulted in an enhancement of DENV-2 infection, as evidenced by the plaque assay (Figure 7).

The postexposure to water extract at its maximum nontoxic concentration of 625 *μ*g/mL had the greatest enhancement effect. The viral titer increased by more than 2 log pfu/mL from log 4.5 pfu/mL to log 7.17 pfu/mL (59%) and from log 4.03 pfu/mL to log 6.16 pfu/mL (53%) following DENV-2 infection with 200 and 2000 pfu, respectively (Figure 7(a)).

The enhancement of DENV-2 replication, observed when C6/36 cells were posttreated with methanol extract, was concentration dependent (Figure 7(b)). The highest effect on viral replication was observed when the highest concentration of methanol extract, 156 *μ*g/mL, was used, resulting in a 2 log increment of the viral titer (49%) in comparison to nontreated virus control. However, when C6/36 cells were infected with 2000 pfu of DENV-2, the extract concentration of 156 *μ*g/mL yielded a significant enhancement of the viral titer from log 4.03 pfu/mL to log 5.85 pfu/mL (45%), while at lower concentration (10 and 39 *μ*g/mL), the enhancement effect was much lower, although this difference was not statistically significant. The results obtained by plaque assay confirmed the previous observations recorded on the severe expression of CPE and the loss of cell monolayer in C6/36 cells (Table 1).

Taken together, the posttreatment assays demonstrated that* H. sibthorpioides* showed variable effects, which depend on the host cells, as noted by the contrasting effects between the two cell lines used in this study.

## 4. Discussion

*H. sibthorpioides* has been traditionally used by the Chinese to treat various infections, including those caused by viruses. Recently,* H. sibthorpioides* was shown to exhibit antiviral activity against hepatitis B virus [8]. In this study, the potential use of this plant to inhibit* in vitro* DENV-2 replication was investigated. Dengue virus is strictly a human pathogen, and no appropriate animal model is available to study its pathogenesis.

Currently, there is no antidengue compound known to be isolated from* H. sibthorpioides*. The plant is known to contain triterpenoidal saponins [5, 10]. Simões et al. [11] reported the elucidation of the mechanism of the antiherpetic (HSV-1) activity of two triterpenoid saponins isolated from Brazilian and Chinese plants. Similarly, Subba Rao et al. [12] demonstrated that triterpenoid saponins, containing acylated *β*-amyrin skeleton, exhibit antiinfluenza activity* in vitro*. In this study, we investigated whether* H. sibthorpioides* water and methanolic extracts could confer protection to cells before or after the initiation of DENV-2 infection. This antiviral analysis was performed on C6/36 and Vero cells as a model of infection in mosquito and mammalian cells, respectively.

The ability of* H. sibthorpioides* to confer protection to the cells before DENV-2 infection was tested by pretreating the cells with* H. sibthorpioides* water and methanol extracts for 5 h prior to viral infection. Protection could be conferred through extracellular mechanisms. The* H. sibthorpioides* extracts might interrupt the interaction of several envelope glycoproteins with cell surface receptors requires for fusion of the virion envelope with a cell plasma membrane [13], resulting in ineffective viral infection.* H. sibthorpioides* extracts presented low to mild prophylactic effects on C6/36 and Vero cells, perhaps due to the presence of various plant alkaloids in the crude extract of* H. sibthorpioides,* which may act synergistically to decrease the effective interaction of the active compounds. Furthermore, active compounds associated with antidengue activities in these extracts may be present at low levels in the noncytotoxic dilution of the extract [14], resulting in partial prophylactic activities.

Interestingly, posttreatment assay was conducted in Vero and C6/36 cells to investigate whether the intracellular activities, such as DENV-2 viral RNA replication or viral protein translation and assembly in infected cells, could be affected. The posttreatment was shown to be more effective in lowering DENV-2 titers than the pretreatment assay. Furthermore, the methanolic extract exerted comparatively higher antiviral effects than the water extract in these cells, as demonstrated by the findings from the light microscopy observations and plaque assay. However, the highest inhibitory activity recorded in this study was less than 50%, possibly due to the presence of an insufficient quantity of true antivirals in the extract to completely inactivate DENV-2 [15]. The fact that some degree of inhibitions was observed with* H. sibthorpioides* extracts may suggest that the extracts contain an active component(s) associated with antidengue activities. It is possible that further isolation of the active constituents in this plant may provide better antiviral activities. Similar findings have been reported by others [16–18], where significant antiviral activity against various viruses was observed when the cells were posttreated with compounds or plant extracts following viral infection. This strategy is of great importance because it could be administered once the virus infection has been established in the cell.

It should be noted that different cell lines were used in this study, which may reflect variations in the way different cell types respond to the plant compounds. In this study, surprisingly posttreatment of DENV-2 infected C6/36 cells with* H. sibthorpioides* extracts not only resulted in enhanced viral replication but also did so in a greater magnitude than the antiviral effects observed with Vero cells. This finding concurs with Muller et al. [19], who demonstrated the enhancement of yellow fever virus (YFV) and DENV-2 replication in posttreatment assay and internalization assay using crude venom and isolated toxins from* Crotalus durissus terrificus*, a South American rattlesnake. In addition, Zandi et al. [20] reported that DENV-2 (NGC strain) infectivity and replication increased when flavone was added during and after virus adsorption by Vero cells. The mechanism of dengue virus enhancement in the current study is currently unknown.

## 5. Conclusion

In conclusion, the present study revealed the variable effects of* H. sibthorpioides* on DENV-2 replication in C6/36 and Vero cells, which had low prophylactic function, mild antiviral activities, and a surprising enhancement effect depending on the host cells and treatment used, thus providing new insights on the biological properties of* H. sibthorpioides*. In the future, a more in-depth study will be necessary to elucidate the mechanisms of inhibition and enhancement involved and to determine whether the inhibitory effect of* H. sibthorpioides* could be expressed fully to reveal more potent antiviral activity, when the purified active compounds are used.

## Figures and Tables

**Table 1:** Morphological changes in DENV-2 infected cells.

Cell line	Solvent	Concentration (*µ*g/mL)	Dilution of extract	Degree of CPE manifestation			
				Pretreatment		Posttreatment	
				200 pfu	2000 pfu	200 pfu	2000 pfu
Vero	Water	20	1/64	+++	+++	+++	+++
		156	1/8	+++	+++	+++	+++
		1250	1/2	+++	+++	+++	+++
	Methanol	39	1/16	+++	+++	+++	+++
		156	1/4	+++	+++	++	++
		625	1/2	+++	+++	++	++

C6/36	Water	10	1/64	+++	+++	++++	++++
		78	1/8	+++	+++	++++	++++
		625	1/2	+++	+++	++++	++++
	Methanol	10	1/16	+++	+++	++++	++++
		39	1/4	+++	+++	++++	++++
		156	1/2	+++	+++	++++	++++

“++++” 100% virus growth-like CPE, “+++” 75% virus growth-like CPE, “++” 50% virus growth-like CPE, “+” <50% virus growth-like CPE, “−” no CPE.
